# Effect of Anxiety on Pain Perception During Infiltration Anesthesia of Maxillary Teeth in a Group of Adult Sudanese Patients at Khartoum Teaching Dental Hospital

**DOI:** 10.7759/cureus.93988

**Published:** 2025-10-06

**Authors:** Mahmoud Y Aldooma, Elneel A Mohamed Ali

**Affiliations:** 1 Department of Oral and Maxillofacial Surgery, Sudan Medical Specialization Board, Khartoum, SDN; 2 Department of Oral and Maxillofacial Surgery, Faculty of Dentistry, University of Khartoum, Khartoum, SDN

**Keywords:** dental anxiety, dental injection, infiltration anesthesia, khartoum teaching dental hospital, pain perception, sudan

## Abstract

Background

This study aimed to assess the effect of dental anxiety on pain perception during palatal infiltration anesthesia in Sudanese patients. This study was conducted at the Khartoum Teaching Dental Hospital from June 1, 2021, to August 30, 2021.

Methodology

Utilizing convenience sampling, 104 adult Sudanese patients were included in this cross-sectional, hospital-based study. Anxiety was assessed using the Modified Dental Anxiety Scale, while pain perception was assessed using the Numeric Rating Scale.

Results

A significant correlation between dental anxiety and pain perception was identified (an increase in dental anxiety increased pain perception, as evidenced by a Pearson coefficient of 0.405. Additionally, dental anxiety was significantly higher in females (10.73) compared to males (8.27), with a p-value of 0.006. Further, local anesthetic injection was the most anxiety-provoking factor in the dental clinic.

Conclusions

These findings emphasize the necessity for dental practitioners to implement tailored strategies addressing the specific anxiety-related concerns of their patients, particularly regarding local anesthetic administration. This approach can enhance patient comfort and potentially improve treatment outcomes by reducing anxiety and pain perception during dental procedures.

## Introduction

Kleinberg and Bromberg described dental anxiety as the apprehension that something terrible will happen with dental treatment or certain aspects of dental treatment [1]. Extensive empirical evidence now substantiates that psychological factors significantly influence pain perception. Neuroimaging investigations reveal that attentional states, emotional valence (both positive and negative), stress, empathy, and the administration of placebo treatments modulate activity within pain pathways. Furthermore, psychological factors such as expectation, attention, and emotional state play a crucial role in modulating pain perception [2]. A range of psychological, social, and sensory factors influences dental anxiety. Early conditioning experiences, particularly negative or traumatic dental encounters during childhood, are among the most significant contributors. Anxiety may also develop through indirect exposure, such as observing anxious family members. Personality traits such as neuroticism and heightened self-awareness, limited understanding of dental procedures, and exposure to distressing portrayals of dentistry in the media can further intensify fear. Additional factors include maladaptive coping styles, distorted perceptions of body image, and the inherent vulnerability associated with reclining in a dental chair [3-6]. Sensory triggers within the dental environment can also provoke anxiety. These include visual stimuli such as needles and air-turbine drills, auditory cues such as drilling sounds and patient distress, olfactory sensations from eugenol and cut dentine, and tactile experiences involving high-frequency vibrations [7,8].

Dental anxiety and the anticipation of pain are correlated with neurophysiological alterations in heart rate, respiratory rate, and blood pressure before, during, and after dental interventions [9]. The management of anxiety in patients undergoing various dental treatments, such as oral and maxillofacial surgery, is critical. Effective anxiety control improves patient well-being and contributes to reduced treatment-related stress [10]. While dental anesthesia constitutes an integral component of patient care, the administration of an injection can evoke anxiety or trepidation, serving as a significant deterrent for patients contemplating dental treatment [11]. Injections (specifically, palatal injections have been identified as the most excruciating), at least from the patients’ perspective, have the potential to elicit dental anxiety or fear in pediatric patients and represent a source of distress or a compelling rationale for adults to eschew dental interventions entirely [12]. Fear of dental injections includes apprehension toward needles and anticipated pain, anxiety surrounding the administration of local anesthesia, particularly concerns about potential side effects, fear of contracting infectious diseases, and apprehension about possible tissue injury [13].

Pain perception is a complex emotional and sensory experience influenced by previous experiences, stress, clinical context, and anxiety. This multifactorial nature has been documented in prior studies [14,15].

Patients may exhibit difficulties in cooperating with dental practitioners when experiencing anxiety during treatment, which may prolong the duration required and elevate the complexity of executing procedures, thereby compromising the quality of treatment outcomes [16].

Numerous studies have examined the influence of anxiety on pain perception across various dental procedures, including implants [17,18], restorative treatments [19], dental hygiene interventions [20], oral health assessments [21], root canal therapy [22], oral surgery [23,24], and periodontal treatment [25,26]. These investigations consistently demonstrate that anxiety significantly impacts pain perception. However, one study reported no such effect [27]. Although the hypothesis that psychological factors, including gender differences, influence dental anxiety has been well-documented in previous literature, there remains a need to explore this phenomenon within specific cultural and clinical contexts, such as among Sudanese patients undergoing oral and maxillofacial procedures.

Thus, this analytical, cross-sectional, hospital-based study aimed to assess the relationship between dental anxiety and pain perception during palatal infiltration anesthesia within a Sudanese cohort at Khartoum Teaching Dental Hospital (KTDH). Because the majority of Sudanese patients receive medical care at KTDH, this facilitated the collection of comprehensive data from patients undergoing dental procedures, as it is crucial to comprehend how dental anxiety affects patients in this specific context to enhance dental care and promote a sense of comfort among patients during their treatments.

## Materials and methods

The study protocol was reviewed and approved by the Institutional Review Board (IRB) of the Sudan Medical Specialization Board (approval number: EA2509000338). The study was conducted at KTDH from June 2021 to August 2021, during which 104 adult Sudanese patients were selected using a convenience sampling methodology. This process involved including all patients seeking extraction of an upper tooth who met the inclusion criteria (i.e., provided informed consent and did not have any medical conditions that could impact the study’s outcomes, such as infections at the injection site or medications affecting pain perception) during the specified timeframe. Upon their arrival at the outpatient department, patients were given a detailed explanation of the study and its objectives while seated in the dental chair, and written consent was subsequently obtained. The consent procedure was meticulously designed to be both clear and comprehensive, thereby facilitating patients’ ability to make an informed decision regarding their participation in the research. The Modified Dental Anxiety Scale (MDAS) [28] is a valid and reliable tool for assessing dental anxiety [29]. Translated into Arabic using Google Translate, it was administered to evaluate the anxiety levels of the participants [30,31]. The reliability and internal consistency of the questionnaire were maintained despite the translation (Cronbach’s alpha of 0.727), suggesting that the translated scale yielded results comparable to the original version.

Subsequently, the patient was posed with the following five questions: how do you feel when you decide to visit your dentist tomorrow? When are you in the waiting room? When is your tooth to be drilled for a filling? When are your teeth to be polished/scaled? When are you about to receive a local anesthetic injection in your gum? Anxiety levels were assessed using a five-point Likert scale, where responses were coded as follows: not anxious = 1, slightly anxious = 2, fairly anxious = 3, very anxious = 4, and extremely anxious = 5. The overall anxiety score was derived from the summation of all five items, ranging from 5 to 25, categorized as follows: <10 (not anxious at all), 10-18 (moderately anxious), and ≥19 (extremely anxious), the latter indicating a highly anxious or dentally phobic patient. Following the anxiety assessment, local anesthetic palatal infiltration was performed using 2% lidocaine with adrenaline 1:200,000, administered by resident house officers using an infiltration technique. Topical anesthesia was not applied. Needle gauge and injection speed were not strictly standardized across operators. This variability is noted and addressed in the limitations. The injection was administered by a resident house officer around the tooth designated for extraction. Patients were subsequently instructed to evaluate their pain perception during the palatal injection utilizing a Numeric Rating Scale (NRS), where they rated their pain from 0 (no pain) to 10 (worst pain).

The data accrued, encompassing both anxiety and pain levels, were documented on a data collection sheet and meticulously cleaned. Statistical analysis was performed using SPSS Statistics for Windows, version 25.0 (IBM Corp., Armonk, NY, USA). Descriptive statistics were employed to describe the data frequency distribution, including tables, as well as the mean and standard deviation. Analysis of variance, t-tests (utilized for comparative analysis among study groups), chi-square tests, and Pearson’s correlation analysis were implemented to evaluate the reliability and correlation of the study parameters across the various study groups. For all statistical tests, a p-value of less than 0.05 was considered statistically significant. Significant values were marked with an asterisk (*) in the tables. The objective of the study was to elucidate the relationship between dental anxiety and pain perception, thereby underscoring the necessity for effective management strategies aimed at enhancing patient comfort during dental procedures.

## Results

The study’s findings demonstrated a moderate correlation between dental anxiety and pain perception (with a Pearson correlation coefficient of 0.405), as seen in Table 1, indicating that an elevated level of anxiety was associated with increased pain perception during dental interventions. Furthermore, it was noted that female participants exhibited a markedly higher level of anxiety (10.73 ± 4.6) compared to males (8.27 ± 3.6) (p = 0.006), as delineated in Table 2.

**Table 1:** Correlation between the Modified Dental Anxiety Scale and the Numerical Rating Scale. The correlation was determined to be statistically significant at the 0.01 level (two-tailed).

N	Pearson correlation	P-value
104	0.405	0.000

**Table 2:** Gender-wise findings of dental anxiety. Values are presented as mean ± standard deviation unless otherwise indicated. P-values <0.05 and <0.001 were considered statistically significant. MDAS = Modified Dental Anxiety Scale; NRS = Numeric Rating Scale

	Gender	N	Mean	SD	P-value
MDAS	Male	37	8.27	3.641	0.006*
	Female	67	10.73	4.647	
NRS	Male	37	4.54	2.795	0.933
	Female	67	4.49	2.743	

The average NRS score for the entire cohort was 4.5 ± 2.8, with a score of 4.5 ± 2.8 for males and 4.5 ± 2.7 for females. The disparity in mean NRS scores between genders was statistically insignificant, as illustrated in Table 2. This finding implies that males may not fully disclose their real levels of anxiety, despite experiencing it. Hence, each patient’s experience is distinct, potentially influenced by various factors beyond mere gender, including personal history, cultural context, and individual coping strategies. Overall, nine (8.7%) patients were extremely anxious before attending, eight (7.7%) during waiting, six (5.8%) when their tooth was about to be drilled, and four (3.8%) when they were about to undergo scaling. Overall, 14 (13.5%) patients who were extremely anxious, the administration of local anesthetic injections was correlated with the highest level of anxiety (Table 3). This observation implies that patients may endure heightened anxiety specifically associated with the delivery of local anesthetics, signifying a salient area for clinicians to focus on to enhance patient comfort during dental procedures. Understanding the origins of this anxiety could facilitate the development of improved management strategies, such as pretreatment counseling or the employment of sedation techniques, ultimately contributing to an enhanced overall patient experience in dental care.

**Table 3:** Anxiety and its provoking factors.

	Not anxious	Slightly anxious	Fairly anxious	Very anxious	Extremely anxious
Before attending	59 (56.7%)	21 (20.2%)	8 (7.7%)	7 (6.7%)	9 (8.7%)
During waiting	49 (47.1%)	23 (22.1%)	13 (12.5%)	11 (10.6%)	8 (7.7%)
Drilling	64 (61.5%)	15 (14.4%)	12 (11.5)	7 (6.7%)	6 (5.8%)
Scaling	69 (66.3% )	18 (17.3%)	6 (5.8%)	7 (6.7%)	4 (3.8%)
Local anesthesia	44 (42.3%)	16 (15.4%)	16 (15.4%)	14 (13.5%)	14 (13.5%)

## Discussion

This study revealed a significant relationship between dental anxiety and pain perception in a Sudanese population. The results demonstrated that the application of the Arabic form of the MDAS was effective in evaluating anxiety levels among Sudanese patients.

Our findings align with previous studies that reported increased pain perception among anxious dental patients [15,17,18]. In contrast, a study by Onwuka et al. found no significant association between anxiety and pain response. This difference in the result of our study could be due to pain-provoking factors in the survey, which was the surgical extraction of the impacted mandibular third molar, which is usually complicated by postoperative pain, regardless of the state of anxiety [32].

Translation of MDAS into the Arabic language was to accurately assess the anxiety levels of the predominantly Arabic-speaking patient population at KTDH and to ensure that patients could accurately understand the questionnaire, thereby allowing them to express their anxiety levels effectively and were able to engage with the material meaningfully and provide informed responses [33]. This Arabic adaptation can be utilized in other Arabic-speaking nations to assess anxiety and pain perception levels.

Moreover, the study revealed that female participants exhibited a markedly higher level of anxiety than males, similar to a study by Dadalt et al. [34]. This emphasizes the significance of male patients who exhibit a non-anxious demeanor, as they may necessitate additional assistance in articulating their anxiety levels accurately, thereby facilitating enhanced management of their dental care experiences. Furthermore, it was established that the administration of local anesthetics constitutes the primary anxiety-inducing factor within the dental environment, indicating that addressing this specific issue through the modification of local anesthetic techniques, such as the application of topical anesthetics before the injection, can alleviate anxiety, diminish pain perception, and ensure favorable treatment outcomes [35]. Future investigations should focus on targeted interventions designed to alleviate dental anxiety and improve patient comfort during dental procedures.

This study has several limitations that warrant consideration. First, its cross-sectional design limits the ability to infer causality between dental anxiety and pain perception. Second, participants were recruited by convenience sampling during the study period (the sample was drawn exclusively from a single dental hospital in Khartoum, KTDH, which may restrict the generalizability of the findings to other populations. Although the total sample (n = 104) provided adequate power to detect moderate effect sizes, the study was underpowered to detect small effects. Therefore, the results should be interpreted cautiously and validated in larger, probabilistically sampled cohorts. Third, reliance on self-reported instruments such as the MDAS introduces potential response bias. Fourth, the specific details of anesthetic technique, such as the speed of injection, the size, and length of the needle, were not uniformly controlled, which may have influenced pain perception and anxiety outcomes. Future studies should standardize and report these variables or include them as covariates. Moreover, confounding variables, including socioeconomic status and cultural attitudes toward pain, were not accounted for and may have influenced the observed associations.

## Conclusions

Addressing dental anxiety is imperative to enhance patient experiences and treatment outcomes, thereby requiring customized strategies that take into account the unique backgrounds and anxiety triggers of individual patients. This study demonstrated a clear association between elevated dental anxiety and increased pain perception during infiltration anesthesia of maxillary teeth among adult Sudanese patients. These findings highlight the need for Sudanese dental practitioners to routinely assess anxiety levels before administering local anesthesia, particularly in public hospital settings where patient apprehension may be heightened due to limited resources and high patient turnover. Integrating simple, cost-effective anxiety-reducing interventions, such as verbal reassurance, patient education, and culturally sensitive communication, could significantly improve patient comfort and procedural outcomes. Moreover, these results underscore the importance of incorporating psychological screening tools such as the MDAS into routine dental assessments to better tailor treatment approaches. Future research should explore longitudinal designs and multi-center studies across Sudan to validate these findings and inform national guidelines for anxiety management in dental care.
